# Wnt5a and CCL25 promote adult T-cell acute lymphoblastic leukemia cell migration, invasion and metastasis

**DOI:** 10.18632/oncotarget.16559

**Published:** 2017-03-25

**Authors:** Xinzhou Deng, Zhenbo Tu, Meng Xiong, Kingsley Tembo, Lu Zhou, Pan Liu, Shan Pan, Jie Xiong, Xiangyong Yang, Jun Leng, Qian Zhang, Ruijing Xiao, Qiuping Zhang

**Affiliations:** ^1^ Department of Immunology, School of Basic Medical Sciences, Wuhan University, Wuhan, Hubei, China; ^2^ Department of Clinical Oncology, Taihe Hospital, Hubei University of Medicine, Shiyan, Hubei, China; ^3^ Department of Hematology, Taihe Hospital, Hubei University of Medicine, Shiyan, Hubei, China; ^4^ Department of Biochemical Engineering, Hubei University of Technology Engineering and Technology College, Wuhan, Hubei, China; ^5^ Department of Hematology, Zhongnan Hospital of Wuhan University, Wuhan, Hubei, China; ^6^ Hubei Provincial Key Laboratory of Developmentally Originated Disease, Wuhan, Hubei, China

**Keywords:** Wnt5a, CCL25/CCR9, T-cell acute lymphoblastic leukemia, PI3K, RhoA

## Abstract

Adult T-cell acute lymphoblastic leukemia (T-ALL) is a refractory leukemia. We previously showed that CCL25/CCR9 promotes T-ALL metastasis. In the present study, we assessed the effects of CCL25 on Wnt expression and the effects of Wnt5a and CCL25 on PI3K/Akt and RhoA activation. Transwell assays and mouse xenograft experiments were utilized to assess the effects of Wnt5a and CCL25 on MOLT4 cell invasion, migration and metastasis. The effects of Wnt5a on MOLT4 cell actin polarization and pseudopodium formation were examined using laser scanning confocal microscopy and scanning electron microscopy. CCL25 induced Wnt5a expression in MOLT4 cells by promoting protein kinase C (PKC) expression and activation. Wnt5a promoted MOLT4 cell migration, invasion, actin polarization, and lamellipodium and filopodia formation via PI3K/Akt-RhoA pathway activation. These effects were rescued by PI3K/Akt or RhoA knockdown or inhibition. Additionally, Wnt5a in cooperation with CCL25 promoted MOLT4 cell mouse liver metastasis and stimulated RhoA activation. These results show that CCL25/CCR9 upregulates Wnt5a by promoting PKC expression and activation in MOLT4 cells. This in turn promotes cell migration and invasion via PI3K/Akt-RhoA signaling, enhancing cell polarization and pseudopodium formation. These findings indicate that the PI3K/Akt-RhoA pathway is likely responsible for Wnt5a-induced adult T-ALL cell migration and invasion.

## INTRODUCTION

T-cell acute lymphoblastic leukemia (T-ALL) is an aggressive leukemia derived from the malignant transformation of T-cell precursors, and about 25% of cases occur in adults [1]. A large number of T-ALL cases relapse or exhibit extramedullary infiltration leading to poor prognosis [2, 3]. The overall T-ALL survival rate is 85% in children, but only 40% in adults [4, 5]. Identifying mechanisms involved in adult T-ALL metastasis may lead to innovative treatment methods and improved patient outcomes.

Chemokines combined with their specific receptors provide directional cues for leukocyte and tumor cell migration, metastasis, entry into the circulation, homing, and colonization [6–10]. Wnt family members and their downstream effectors may promote tumorigenesis-related processes, including tumor cell proliferation, differentiation and metastasis [11–13]. Protein kinase C (PKC) phosphorylation is reportedly involved in CXCL12-induced Wnt5a upregulation in T cells [14], and Wnt may play a role in chemokine-triggered tumorigenesis [15–18].

CC chemokine ligand 25 (CCL25), together with its receptor CC chemokine receptor 9 (CCR9), induces chemotaxis in immature CD4^+^/CD8^+^ and mature CD4^+^ or CD8^+^ thymocytes, suggesting that CCL25/CCR9 stimulates T-cell migration in the thymus [19]. CCR9 expression is selectively increased on CD4^+^ T cells in T-ALL, inducing chemotactic migration by T-ALL CD4^+^ cells [20, 21].

We previously showed that CCL25 induces metastasis in CCR9-overexpressing MOLT4 cells via the RhoA-ROCK-MLC axis [22] and promotes pseudopodium formation and ERM protein family translocation from the cytoplasm to the cell membrane [23], suggesting that RhoA is associated with CCL25-induced T-ALL metastasis. However, the relationship between CCL25/CCR9 and Wnt signaling pathways in adult T-ALL has not yet been elucidated.

Wnt5a is a noncanonical member of the Wnt protein family and promotes oncogenesis and tumor metastasis [24, 25]. Wnt5a triggers Wnt/planar cell polarity (PCP) pathway signaling by activating small Rho-GTPases, such as RAC1 [26] and RhoA [27], which increases cytoskeleton reorganization and cellular polarity during development and tissue homoeostasis [28]. Wnt5a reportedly activates RhoA through Dvl2-Daam1 [24] and PI3K/Akt pathway [29] activation. PI3K/Akt pathway activation is linked to tumorigenesis in a wide variety of human cancers [30, 31], including T-ALL [32].

In this study, we investigated the relationship between CCL25/CCR9 and Wnt family members in adult T-ALL, focusing on the Wnt proteins stimulated by CCL25/CCR9 signaling. Our results shed light on the mechanisms by which CCL25/CCR9 and Wnt promote metastasis in adult T-ALL.

## RESULTS

### CCL25 promotes Wnt5a expression in MOLT4 cells

MOLT4 cells naturally overexpress CCR9, the unique receptor of CCL25 [33], and CD7 is a MOLT4 cell detection marker [34]. We detected CCR9 expression and human CD7 via flow cytometry. 81.9% of MOLT4 cells expressed high levels of CCR9, and 72.1% expressed CD7 (Supplementary Figure 1).

**Figure 1:**
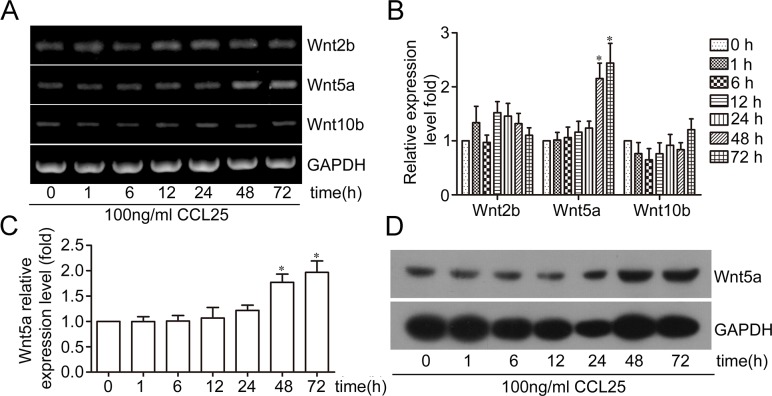
Effect of CCL25/CCR9 on Wnt5a expression in MOLT4 cells MOLT4 cells were treated with 100 ng/ml CCL25 at the indicated times, and Wnt expression was detected via RT-PCR **(A)** Wnt mRNA relative expression was calculated by optical density (OD) values normalized to GAPDH **(B)** qPCR and western blotting were used to detect Wnt5a expression **(C)** & **(D)** Data are presented as means ± SD of 3 independent experiments. *p<0.05.

To investigate the effect of CCL25/CCR9 on Wnt expression, MOLT4 cells were exposed to 100 ng/ml CCL25. RT-PCR results showed that of the 18 Wnt members, only Wnt2b, Wnt5a, and Wnt10b were expressed in MOLT4 cells. Wnt2b levels were slightly upregulated after exposure to CCL25 at 1, 12, 24, and 48 h, with a maximum 1.5-fold increase at 12 h. Wnt5a levels were increased after 48 and 72 h CCL25 treatment, by 2.15- and 2.44-fold, respectively. Wnt10b expression was slightly downregulated after 6 h CCL25 treatment, and slightly upregulated at 72 h treatment (Figure 1A–1B). Real time PCR and western blotting were also used to examine Wnt5a expression, and we observed that Wnt5a was upregulated in MOLT4 cells after treatment with CCL25 for 48 and 72 h (Figure 1C–1D). These results showed that CCL25 induced Wnt5a expression in MOLT4 cells.

### CCL25 promotes Wnt5a expression via PKC upregulation and activation

**Figure 2:**
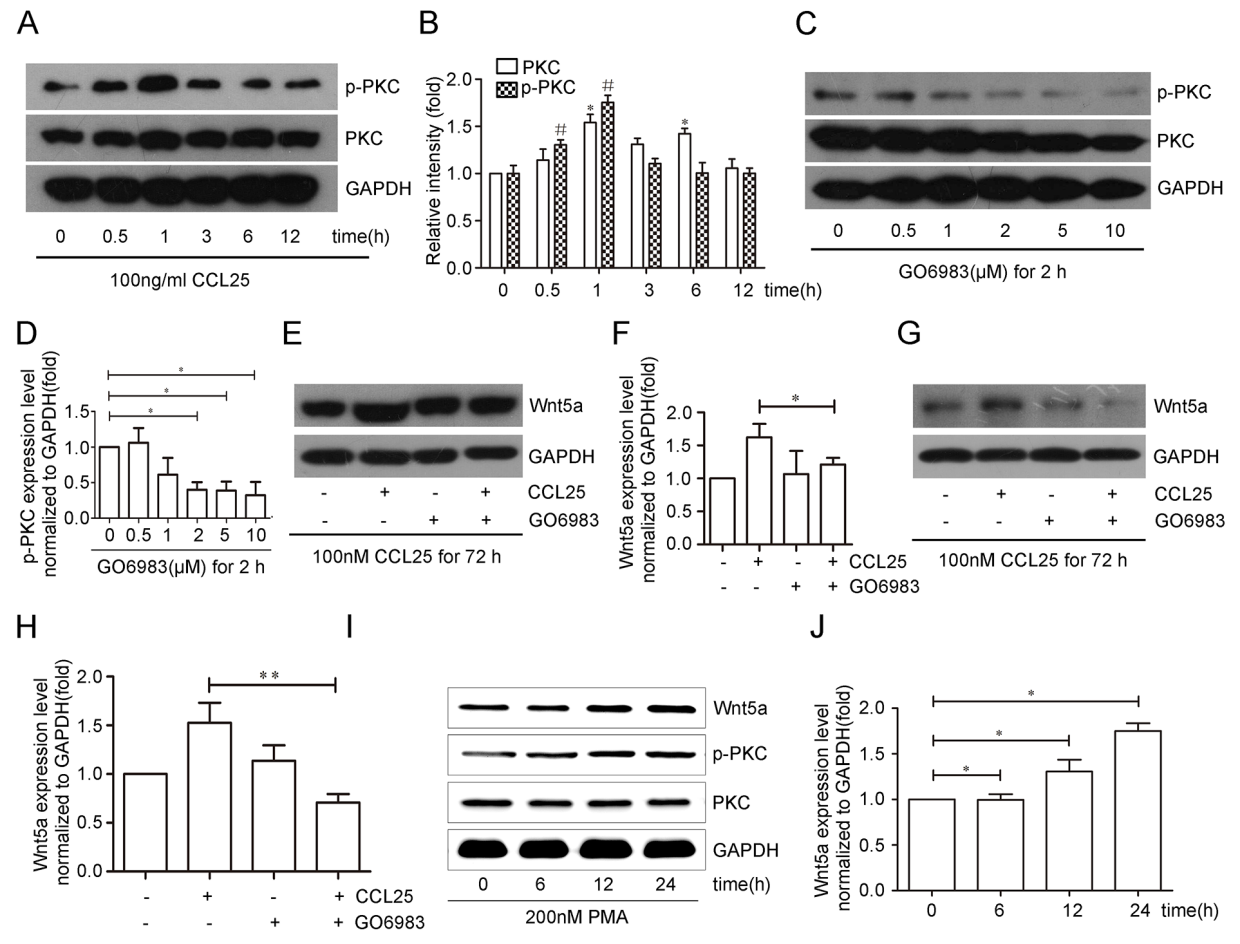
CCL25 promotes Wnt5a expression via PKC upregulation and phosphorylation in MOLT4 cells MOLT4 cells were treated with 100 ng/ml CCL25 at the indicated times, and PKC expression and phosphorylation were analyzed **(A)** p-PKC and PKC (total PKC) levels were normalized to that of PKC (total PKC) and GAPDH respectively **(B)** MOLT4 cells were treated with different concentrations of the PKC inhibitor, GO6983 for 2 h, and PKC phosphorylation inhibition was analyzed by western blot **(C)** PKC phosphorylation levels normalized to that of PKC (total PKC) **(D)** MOLT4 cells were treated with 2 μM GO6983 for 2 h prior to CCL25 treatment (100 ng/ml, 72 h), and Wnt5a levels were analyzed via western blot **(E)** Wnt5a levels were normalized to that of GAPDH **(F)** MOLT4 cells were treated with 5 μM GO6983 for 2 h prior to CCL25 treatment (100 ng/ml, 72 h), and Wnt5a levels were analyzed via western blot **(G)** Wnt5a levels were normalized to that of GAPDH **(H)** MOLT4 cells were treated with 200 nM PMA at the indicated times, and Wnt5a levels were analyzed by western blot **(I)** Wnt5a levels were normalized to that of GAPDH **(J)** Data are presented as means ± SD of 3 independent experiments. *p<0.05, **p<0.01.

We investigated the molecular mechanism by which CCL25 stimulated Wnt5a expression in MOLT4 cells, and assessed the role of PKC in this process. PKC expression and phosphorylation were analyzed in MOLT4 cells treated with CCL25. PKC was upregulated after being treated with CCL25 at 1 and 6 h, and then dropped back to baseline levels after 12 h. However, the level of phosphorylated PKC increased within 30 min of CCL25 treatment, with maximal levels at 1 h, and then dropped back to baseline levels after 3 h (Figure 2A-2B).

PKC was inhibited to determine whether it was required for CCL25-induced Wnt5a expression. Efficiency of the PKC inhibitor, GO6983, was examined by western blotting (Figure 2C-2D). MOLT4 cells were treated with 2 μM or 5 μM GO6983 for 2 h prior to CCL25 treatment, and then analyzed for Wnt5a expression by western blotting. In the presence of 2 μM or 5 μM GO6983, Wnt5a protein induced by CCL25 was reduced by 26% (Figure 2E–2F) and 57% (Figure 2G–2H), respectively.

Finally, the PKC activator, PMA, was used to analyze the effects of PKC on the Wnt5a expression. MOLT4 cell treatment with 200 nM PMA increased Wnt5a expression after 12 and 24 h (Figure 2I–2J). These results suggest that CCL25-induced Wnt5a expression occurs via PKC upregulation and phosphorylation in MOLT4 cells.

### Wnt5a promotes adult T-ALL cell migration and invasion

**Figure 3:**
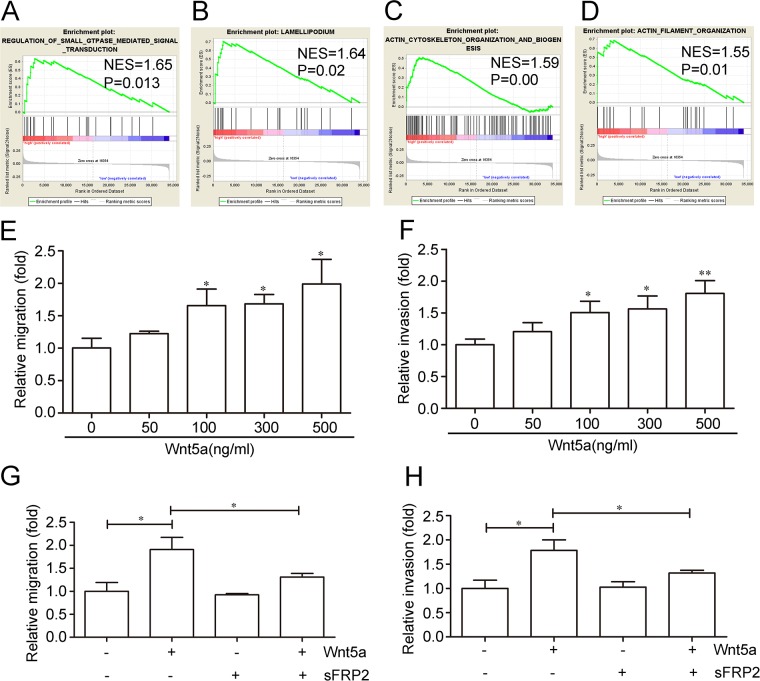
Effects of Wnt5a on adult T-ALL cell migration and invasion Wnt5a is associated with several migration-related biological processes and signaling pathways in the adult T-ALL GEO dataset, GSE42328, as shown by Gene Set Enrichment Analysis (GSEA) **(A**–**D)** Relative Wnt5a-induced MOLT4 cell migration and invasion rates as determined by transwell or matrigel-transwell assays **(E** & **F)** Relative cell migration and invasion rates with or without 12 h of 500 ng/ml Wnt5a treatment following a 1-h pre-incubation in 1000 ng/ml Wnt5a inhibitor (sFRP2) **(G** & **H)** Data are presented as means ± SD of 3 independent experiments. *p<0.05, **p<0.01.

The adult T-ALL GEO dataset, GSE42328, was used to analyze the possible functions of Wnt5a in adult T-ALL by GSEA. GSEA analysis results showed that many biological processes and signaling pathways were associated with Wnt5a (Supplementary Table 1). Several migration-related biological processes and signaling pathways were enriched in the Wnt5a “high” group in adult T-ALL GEO datasets, such as regulation of small GTPase-mediated signal transduction (Figure 3A), lamellipodium formation (Figure 3B), actin cytoskeleton organization and biogenesis (Figure 3C), and actin filament organization (Figure 3D). Our study focused on the effects of Wnt5a on cell migration and invasion in adult T-ALL.

The adult T-ALL MOLT4 cell line was treated with different doses of Wnt5a, the migration and invasion rates were examined by transwell and matrigel transwell assays. We found that 500 ng/ml Wnt5a treatment stimulated an approximately 1.92-fold increase in MOLT4 cell migration (Figure 3E), and enhanced invasion by approximately 1.81-fold compared to controls (Figure 3F). Furthermore, pre-incubation with 1000 ng/ml Secreted Frizzled-related protein 2 (sFRP2), an antagonist that directly binds Wnt [35], abolished Wnt5a-enhanced MOLT4 cell migration (Figure 3G) and invasion (Figure 3H). These results indicated that Wnt5a may promote adult T-ALL cell migration and invasion.

### Wnt5a induces MOLT4 cell migration and invasion via RhoA activation

**Figure 4:**
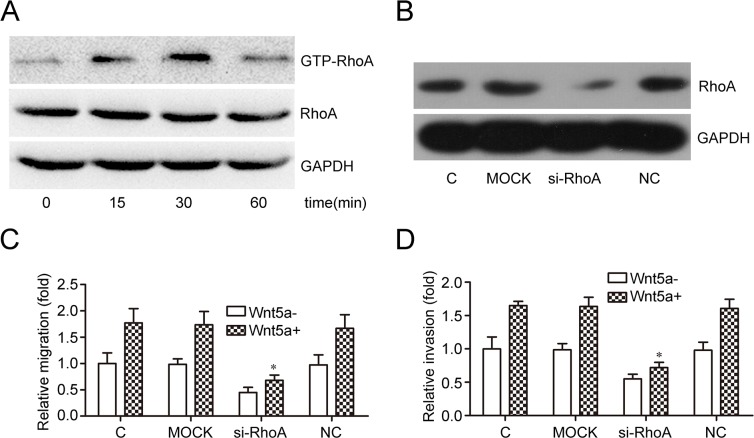
Effects of RhoA on Wnt5a-induced MOLT4 cell migration and invasion MOLT4 cells were treated with Wnt5a (500 ng/ml) at the indicated times to assess the effects of Wnt5a on RhoA activation **(A)** Western blotting confirmed RhoA knockdown via siRNA in MOLT4 cells **(B)** Transwell **(C)** and matrigel-transwell **(D)** assays assessed RhoA knockdown on Wnt5a-induced (500 ng/ml for 12 h) MOLT4 cell migration (C, control). Data are presented as means ± SD of 3 independent experiments. *p<0. 05.

GSEA results suggested that Wnt5a may be associated with regulation of small GTPase-mediated signal transduction in adult T-ALL. RhoA is an important member of the small GTPase family, and we assessed the effects of Wnt5a on RhoA activation in MOLT4 cells. Wnt5a (500 ng/ml) stimulated RhoA activation after 15 min, with maximal activation after 30 min (Figure 4A). RhoA silencing via siRNA (Figure 4B) reduced Wnt5a-induced MOLT4 cell migration (Figure 4C) and invasion (Figure 4D). These data suggest that Wnt5a induced MOLT4 cell migration and invasion via activation of RhoA.

### PI3K/Akt is required for Wnt5a-induced RhoA activation, cell migration and invasion

**Figure 5:**
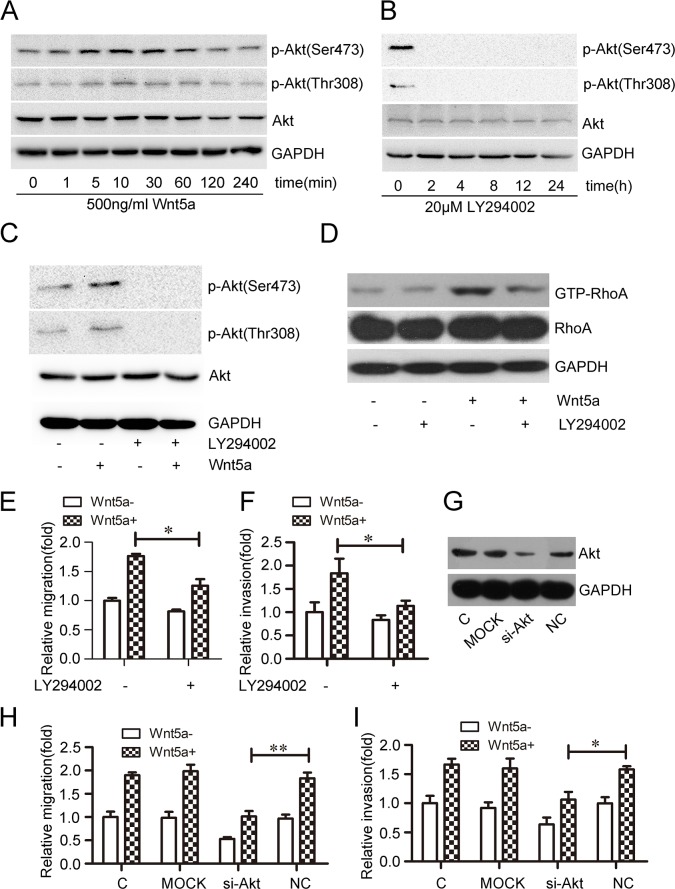
Effect of PI3K/Akt on Wnt5a-induced RhoA activation and MOLT4 cell migration and invasion Wnt5a promoted Akt phosphorylation at Ser473 and Thr308 in MOLT4 cells treated with 500 ng/ml Wnt5a, as detected by western blot **(A)** Conversely, LY294002 inhibited Akt phosphorylation at Ser473 and Thr308 in MOLT4 cells **(B)** LY294002 (20 μM) also inhibited Wnt5a-induced (500 ng/ml) Akt phosphorylation at Ser473 and Thr308 **(C)**, and Rho activation **(D)** in MOLT4 cells. Transwell and matrigel-transwell assays assessed the effects of LY294002 on Wnt5a-induced MOLT4 cell migration **(E)** and invasion **(F)** Western blotting confirmed Akt knockdown via siRNA in MOLT4 cells **(G)** Transwell and matrigel-transwell assays assessed the effects of Akt knockdown on Wnt5a-induced MOLT4 cell migration **(H)** and invasion **(I)** (C, control). Data are presented as means ± SD of 3 independent experiments. *p<0.05, **p<0.01.

Akt phosphorylation generally reflects PI3K activation [36]. Phosphorylated Akt (p-Akt, Ser473 and Thr308 sites), an important signaling molecule downstream of PI3K, was used to measure PI3K activity [37, 38]. To assess the effects of Wnt5a on PI3K/Akt activation, MOLT4 cells were treated with 500 ng/ml Wnt5a at different time points. Akt phosphorylation increased after 5 min, with maximal phosphorylation at 10 min (Figure 5A). The effect of LY294002, a broad spectrum PI3K inhibitor, on Akt phosphorylation was also assessed in MOLT4 cells at different time points. Akt phosphorylation at Ser473 and Thr308 was completely inhibited after 2 h treatment with 20 μM LY294002 (Figure 5B). LY294002 also completely blocked Wnt5a-induced Akt phosphorylation at Ser473 and Thr308 (Figure 5C), and inhibited Wnt5a-induced RhoA activation (Figure 5D).

To examine the effects of PI3K/Akt on Wnt5a-induced cell migration, MOLT4 cells were pre-treated with 20 μM LY294002 for 2 h. Pre-treatment reduced Wnt5a-induced MOLT4 cell migration (Figure 5E) and invasion rates (Figure 5F). Akt silencing via siRNA (Figure 5G) also inhibited Wnt5a-induced MOLT4 cell migration (Figure 5H) and invasion (Figure 5I). This analysis demonstrated that PI3K/Akt signaling is required for Wnt5a-induced RhoA activation and MOLT4 cell migration and invasion.

### Wnt5a induces actin polarization and pseudopodium formation via PI3K/Akt and RhoA activation

**Figure 6:**
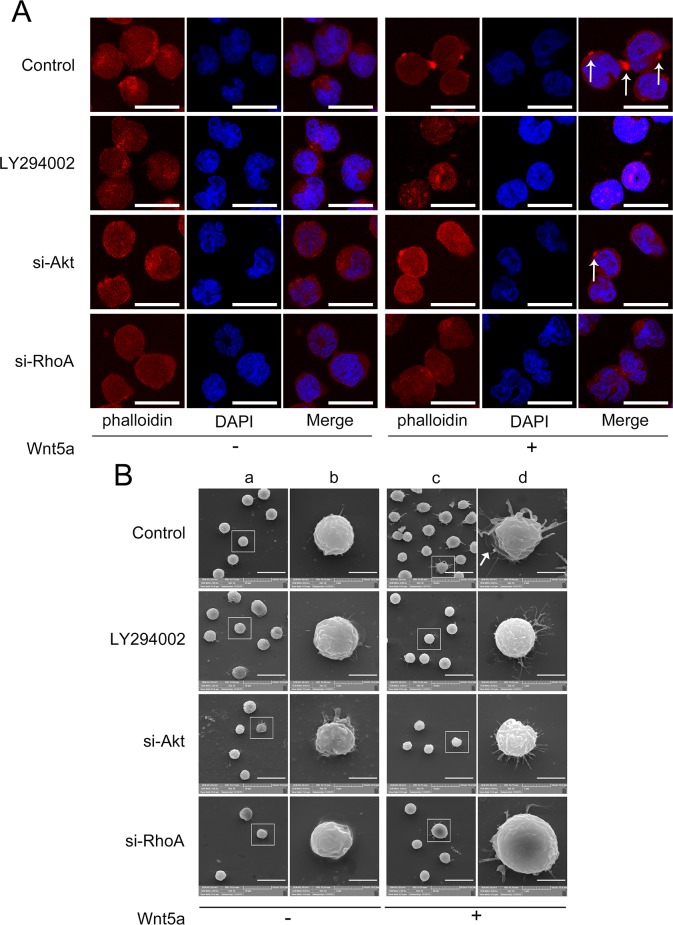
Effects of PI3K/Akt and RhoA on Wnt5a-induced actin polarization and pseudopodium formation MOLT4 cells transfected with Akt siRNA, RhoA siRNA, or scramble (NC) siRNA for 72 h, or pretreated with 20 μM LY294002 for 2 h, were treated with 500 ng/ml Wnt5a for 3 h, and cell polarization was analyzed by LSCM **(A)** Arrows indicate actin patches; scale bars represent 10 μm. Pseudopodia were examined by SEM **(B)** Panel **(b)** is the amplification of **(a)**; **(d)** is the amplification of **(c)**. Scale bars represent 20 μm in (a) and (c) and 5 μm in (b) and (d). Arrow indicates lamellipodium. Each experiment was repeated three times.

GSEA results suggested that Wnt5a might be associated with actin cytoskeleton organization processes in adult T-ALL. We observed that 500 ng/ml Wnt5a treatment promoted actin polarization, while Akt or RhoA silencing (interference rates detection was showed in Supplementary Figure 2) or LY294002 treatment reduced Wnt5a-induced actin polarization (Figure 6A & Supplementary Figure 3). These data demonstrate that PI3K/Akt and RhoA may be required for Wnt5a-induced actin polarization.

GSEA results also suggest that Wnt5a is associated with lamellipodium formation in adult T-ALL. We found that Wnt5a not only induced lamellipodium formation, but also induced needle-shaped filopodia formation in MOLT4 cells (Figure 6B). Similar filopodia were also found in embryonic *Drosophila* primary neurons by Goncalves, *et al*. [39]. LY294002 treatment, or Akt or RhoA silencing via siRNA reduced Wnt5a-induced lamellipodium and filopodia formation. Furthermore, we found that Wnt5a-induced filopodia were more slender in Akt silenced, RhoA silenced and Akt inhibited groups compared to controls. Our results suggested that PI3K/Akt and RhoA activation are essential for Wnt5a-induced lamellipodium and filopodia formation in MOLT4 cells.

### Wnt5a cooperates with CCL25 to promote MOLT4 cell metastasis

**Figure 7:**
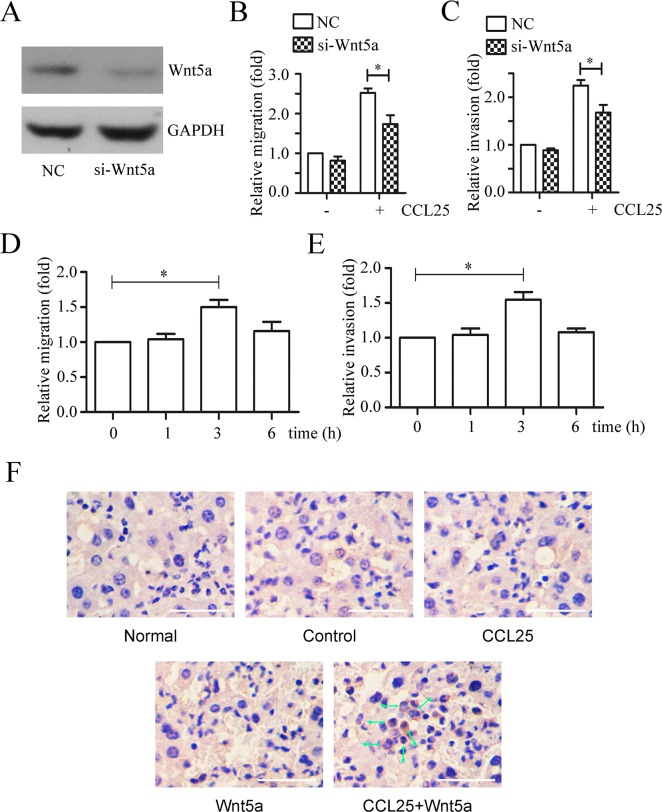
Effects of Wnt5a on CCL25-induced MOLT4 cell metastasis Western blotting confirmed Wnt5a knockdown via siRNA in MOLT4 cells (NC, scramble siRNA) **(A)** Scramble (NC) or Wnt5a siRNA-transfected MOLT4 cells were subjected to transwell and matrigel-transwell assays in the presence 100 ng/ml CCL25 **(B** & **C)** MOLT4 cells pre-treated with 500 ng/ml Wnt5a at the indicated times were subjected to transwell and matrigel-transwell assays in the presence 100 ng/ml CCL25 **(D** & **E)**. Immunohistochemical staining detected MOLT4 cell infiltration in mouse livers **(F)** Arrows indicate MOLT4 cells; bars represent 100 μm. Data are presented as means ± SD of 3 independent experiments. *p<0.05.

We found that Wnt5a silencing via siRNA (Figure 7A), reduced migration (Figure 7B) and invasion (Figure 7C) induced by CCL25 treatment in MOLT4 cells. Additionally, MOLT4 cell pre-treatment with Wnt5a increased CCL25-induced migration (Figure 7D) and invasion (Figure 7E).

To determine whether Wnt5a can increase CCL25 induced MOLT4 cell migration *in vivo*, mouse tail vein metastasis assays were performed. Xenografted SCID mice were anaesthetized 72 d post-inoculation, and MOLT4 cells in bone marrow and PBMCs were detected via flow cytometry (Supplementary Figure 4). Livers, lungs and spleens were removed, and organs were photographed (Supplementary Figure 5). MOLT4 cell numbers in bone marrow Wnt5a-treated animals were lower than in controls, while MOLT4 cells in peripheral blood of CCL25-, Wnt5a-, and CCL25+Wnt5a-treated animals were reduced compared to controls. However, an *in vitro* cell proliferation assay showed no differences between Wnt5a- or CCL25-treated MOLT4 cells (Supplementary Figure 6). Immunohistochemical staining was used to detect MOLT4 cell infiltration in mouse livers and lungs. Only CCL25+Wnt5a-treated animals exhibited MOLT4 cell metastasis to the liver (Figure 7F), with no metastasis to the lung (data not show). Taken together, our data suggest that Wnt5a alone or in combination with CCL25 promotes adult T-ALL metastasis.

### Wnt5a enhances CCL25-induced RhoA activation

**Figure 8:**
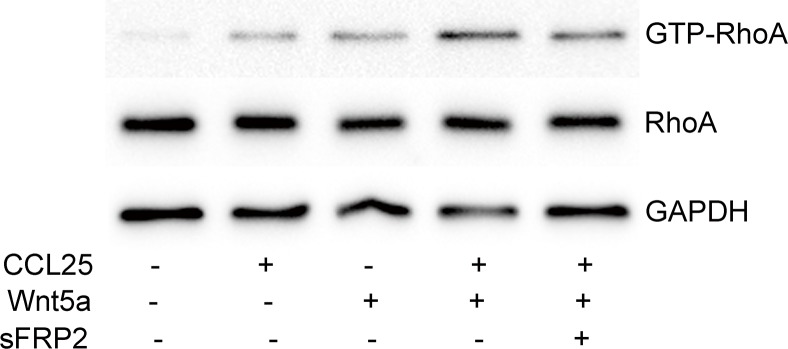
Effect of Wnt5a on CCL25-induced RhoA activation in MOLT4 cells MOLT4 cells were treated with 1000 ng/ml sFRP2 or DMSO for 1 h, followed by 100 ng/ml CCL25 and/or 500 ng/ml Wnt5a for 30 min. Data are presented as means ± SD of 3 independent experiments.

Our previous study found that CCL25 promoted T-ALL cell metastasis via RhoA activation [22], and the present work showed us that RhoA activation is involved in Wnt5a-induced MOLT4 cell migration and invasion. Here, we investigated the effect of Wnt5a on CCL25-induced RhoA activation, and observed that Wnt5a enhances CCL25-stimulated RhoA activation in MOLT4 cells (Figure 8). Our results indicate that Wnt5a cooperates with CCL25 to promote MOLT4 cell metastasis by enhancing CCL25-induced RhoA activation.

## DISCUSSION

Wnt family members and their receptors are associated with tumorigenesis in multiple cancers [40]. CXCL12/CXCR4 signaling promotes cholangiocarcinoma progression and metastasis via the canonical Wnt pathway [41], and Wnt5a is a critical mediator of human and murine T cell CXCL12/CXCR4 signaling and migration [14]. Hu, *et al*. demonstrated that SDF-1/CXCR4 promotes colorectal cancer epithelial-mesenchymal transition and progression by activating Wnt/β-catenin signaling[15]. CCR9 overexpression is also possibly correlated with invasiveness in response to CCL25 in T-ALL [20], prostate cancer [42], breast cancer [43], and melanomas [44]. Through our investigation of the relationship between CCL25/CCR9 and Wnt, we found that CCL25 induced Wnt5a expression via PKC upregulation and phosphorylation in MOLT4 cells.

T-ALL leukemia cell metastasize to other organs is the main causes of recurrence and treatment failure of T-ALL [1]. The mechanisms of T-ALL metastasis is anfractuous. In this study, we found that CCL25/CCR9-Wnt5a pathway maybe involved in adult T-ALL metastasis. Our data showed that Wnt5a knockdown decreased CCL25-induced MOLT4 cell metastasis *in vitro*, it did not fully inhibit CCL25-induced migration and invasion to baseline. This suggests that other mechanisms may be involved in CCL25-induced T-ALL cell metastasis. The CCL25/CCR9 axis reportedly enhances cell proliferation, invasion, and drug resistance via β-catenin activation in pancreatic cancer cells [45], suggesting that β-catenin signaling may also be involved in T-ALL cell migration and invasion. In xenograft experiments, we found fewer MOLT4 cells in bone marrow of Wnt5a-treated animals compared to controls. Similarly, we observed fewer MOLT4 cells in peripheral blood of CCL25-, Wnt5a-, and CCL25+Wnt5a-treated animals, implying that CCL25 and Wnt5a may influence MOLT4 cell proliferation. However, our *in vitro* proliferation assay showed no difference between CCL25- or Wnt5a- treated MOLT4 cells. Our data showed that CCL25 and Wnt5a changed MOLT4 cell distribution in bone marrow and liver in some extent, but this is not the unique mechanism, several other pathway have been proved to related with T-ALL metastasis, such as Notch1 pathway [46], Notch3 pathway [47], IL-7/IL-7R signaling [48] and CCL19/CCR7 signaling [49].

Wnt5a, which belongs to the Wnt family of cysteine-rich secreted glycoproteins [50], participates in both normal development and tumorigenesis via autocrine and paracrine routes [51]. Wnt5a is ubiquitously expressed in morphologically and functionally different populations of cells in bone marrow [52]. Wnt5a expression is downregulated via aberrant methylation in most acute leukemia cases, and is upregulated in non-malignant hematopoietic (NMH) and complete remission (CR) cases; thus, increased Wnt5a expression might act as a tumor suppressor in leukemia [53–56]. However, Wnt5a has also been shown to increase survival in B-cell precursor acute lymphoblastic leukemic Nalm-16 cells [57], and promotes proliferation and migration in HTLV-1-infected adult T-cell leukemia cells [58]. Although Wnt5a expression was downregulated in chronic lymphoblastic leukemia (CLL), Wnt5a-positive CLL cells exhibit increased motility [59]. Our GSEA analysis results showed that several migration-related biological processes were enriched in Wnt5a “high” expressing adult T-ALL samples, including regulation of small GTPase-mediated signal transduction, lamellipodium formation, actin cytoskeleton organization and biogenesis, and actin filament organization. These results were substantiated by transwell and matrigel-transwell assays and xenograft experiments, which showed that Wnt5a promoted adult T-ALL MOLT4 cell migration, invasion, and metastasis. However, our results need to be confirmed in additional adult T-ALL cell lines and primary cells, and the specific role of Wnt5a in MOLT4 cell metastasis must still be investigated, and whether Wnt5a is related with T lymphomas metastasis is not clear, which is also worthy to be investigated.

PI3K/Akt pathway signaling promotes cell survival, cell cycle progression and metastasis in tumors [60]. RhoA, an important Rho family member, is located downstream of p110α, the phosphatidylinositide 3-OH kinase (PI3K) catalytic subunit, which may control endothelial cell migration by regulating RhoA activity [36]. We investigated the effects of PI3K/Akt-RhoA signaling on Wnt5a-induced adult T-ALL cell migration, invasion, polarization and pseudopodium formation *in vitro*. Our results indicate that Wnt5a induced Akt and RhoA activation, actin polarization and pseudopodium formation, and these effects were abrogated by LY294002, a PI3K inhibitor. Liu, *et al*. reported that Wnt5a promoted gastric cancer cell migration via PI3K/Akt/GSK3β/RhoA signaling [29]. We also observed that Akt and RhoA knockdown via siRNA, or treatment with LY294002, suppressed Wnt5a-induced MOLT4 cell migration and invasion. These findings indicate that the PI3K/Akt-RhoA pathway is likely responsible for Wnt5a-induced adult T-ALL cell migration and invasion.

## MATERIALS AND METHODS

### Cells and reagents

Adult T-ALL MOLT4 cells, which express high endogenous CCR9 levels, was purchased from ATCC and cultured in RPMI 1640 medium (Gibco Invitrogen, Paisley, Scotland) containing 10% heat-inactivated fetal bovine serum (FBS), 100 U/ml penicillin, and 100 μg/ml streptomycin. Cells were cultured under humidified conditions at 37°C with 5% CO_2_. Reagents used for cell treatments included recombinant human CCL25 (PeproTech, Rocky Hill, NJ); recombinant Wnt5a protein (rWnt5a) and the Wnt inhibitor, recombinant sFRP2 (R&D Systems, MN); phorbol-12-myristate-13-acetate (PMA) (Sigma, USA); PKC inhibitor GO6983 (Selleckchem, USA); and PI3K inhibitor LY294002 (Cayman Chemical, USA). The following primary antibodies were used: mouse anti-GAPDH (PMK Biotechnology Co., Ltd., China); rabbit anti-Wnt5a (Millipore, Germany); rabbit anti-PKC (anti-PKC alpha) (Cell Signaling Technology, MA); rabbit anti-PKC (pan) (βIIser660) (Cell Signaling Technology, MA); rabbit anti-Akt (Biosynthesis Biotechnology, Beijing, China); rabbit anti-phospho-Akt (Ser473) and rabbit anti-phospho-Akt (Thr308) (Biosynthesis Biotechnology, Beijing, China); and rabbit anti-RhoA (Bioworld Technology, Louis Park, MN). For western blotting, protein bands were detected by incubating membranes with horseradish peroxidase-conjugated secondary antibodies (Antgene Biotech co., LTD., China) and visualized with ECL reagent (Thermo Scientific, Rockford, IL).

### Small interfering RNA (siRNA) transfection

For gene knockdown experiments, we used siRNA duplexes specific for Wnt5a (5′-GGUCGCUAGGUAUGAAUAA TT-3′), Akt (5′-GG AGGGUUGGCUGCACAAA TT-3′, 5′-CUUCUCCG UAGCAGAAUGC TT-3′, and 5′-CUGGAGGCCAAG AUACUUC TT-3′ [29]), and RhoA (On-Target Plus: 5′-GACAUGCUUGCUCAUAGUC TT-3′ [61]) purchased from RIBOBIO (Guangzhou, China). siRNAs were transfected into MOLT4 cells using Lipofectamine 3000 reagent (Invitrogen, Carlsbad, CA) in serum-free OPTI-MEM according to the manufacturer's instructions. Gene expression inhibition efficiency was assessed by western blotting.

### RNA extraction and PCR

Total RNA was isolated using TRIzol reagent (Invitrogen, USA) and cDNA was synthesized using a reverse transcriptase kit (Promega, Madison, WI, USA) according to the manufacturer's protocol. 2×ES Taq MasterMix was used for RT-PCR based detection with the My Cycler™ instrument (Bio-Rad, USA). Real-time quantitative PCR (qPCR) analyses were performed using SYBR Premix Ex Taq™ (Takara, Japan) with the iQTM5 instrument (Bio-Rad, USA). Primer sequences are provided in Supplementary Table 2. Relative mRNA expression was normalized to human GAPDH using the OD value or 2^−ΔΔCt^ method.

### Cell migration and invasion assays

Cell migration and invasion were assessed in a modified Boyden chamber (pore diameter, 8.0 μm) (Costar, Cambridge, MA). For the transwell assay, transwell chambers were coated with 50 μl of 1:3 diluted matrigel. MOLT4 cells were centrifuged and suspended in serum-free culture medium supplemented with 0.1% BSA. 2×10^5^ cells in 100 μl were added to upper wells. Serum free medium containing indicated concentration of Wnt5a or 100 ng/ml CCL25 was added to the lower compartments of the Boyden chamber and cells were allowed to migrate for 12 h at 37°C. Cells that migrated to the lower compartments of the Boyden chamber were counted under a light microscope with a blood counting chamber.

### Pulldown assays

Cell lysate protein concentrations were measured before the pull-down assay using the protein assay kit (Thermo Scientific, Rockford, IL, USA). For detection of active RhoA (GTP-RhoA), equal amounts of protein were incubated with rhotekin Rho-binding peptide immobilized on agarose beads for 45 min at 4°C to activate GTP-Rho bound to rhotekin-agarose. Beads were then washed three times with lysis buffer, resuspended in Laemmli buffer, boiled for 5 min, and subjected to western blotting.

### Western blot

MOLT4 cells were washed twice with PBS, then lysed with ice-cold RIPA lysis buffer (50 mM Tris-HCl pH 7.6, 150 mM NaCl, 2 mM EDTA, 2 mM EGTA, 0.1% Triton-X) containing PMSF protease inhibitor. Lysates were obtained by centrifugation at 12000 g for 10 min at 4°C. Total protein concentrations were measured using the BCA protein assay kit (Thermo Scientific, Rockford, IL, USA) with a PerkinElmer 2030 VICTOR X Multilabel Plate Reader. Whole cell lysates were boiled at 100°C for 5 min in equal volumes of loading buffer (0.5 M Tris-HCl pH 6.8, 2% SDS, 0.05% bromphenol-blue, 20% 2-mercaptoethanol, and 10% glycerol). All sample protein extracts were then separated by 10% SDS-PAGE and transferred to PVDF membranes. After blocking for 2 h in TBST containing 5% non-fat milk, membranes were incubated with primary antibodies diluted in TBST containing 5% non-fat milk at 4°C overnight. Membranes were then washed three times with TBST and incubated with HRP-conjugated secondary antibodies for 2 h at room temperature. They were then washed three times with TBST and signals were detected using an enhanced chemiluminescence detection kit (Thermo Scientific, Rockford, IL, USA).

### Gene set enrichment analysis (GSEA)

GSEA was performed by the JAVA program (http://www.broadinstitute.org/gsea) using the MSigDB C5 collection (GO gene sets) (http://software.broadinstitute.org/gsea/downloads.jsp). Adult T-ALL patient gene profiling data (the GSE42328 series, including 53 adult T-ALL samples) was obtained from the Gene Expression Omnibus (GEO) site (http://www.ncbi.nlm.nih.gov/geo/query/acc.cgi?acc=GSE42328) [62]. Patients were classified into two groups according to Wnt5a expression (top 25%: high vs. bottom 75%: low). Significantly enriched (p<0.05) biological pathways were identified.

### Laser scanning confocal microscopy (LSCM)

To observe actin polarization, MOLT4 cells were transfected with Akt siRNA, RhoA siRNA, or scramble siRNA for 72 h, or pretreated with 20 μM LY294002 for 2 h. Cells were then plated on poly-L-lysine coated glass slides, treated with 500 ng/ml Wnt5a for 3 h, then washed with PBS three times. Subsequently, cells were fixed in 4% paraformaldehyde in PBS for 20 min. After three PBS washes, cells were blocked in PBS containing 3% BSA for 1 h at room temperature, then stained with Phalloidin-Tetramethylrhodamine Conjugate (AAT Bioquest Inc, CA) at room temperature for 2 h. Cells were then washed with PBS and stained with 4′-6-diamidino-2-phenylindole (DAPI) (Southern Biotech, Birmingham, AL) for 3 min at room temperature. A laser scanning confocal microscope (Leica, Germany) was used to examine actin polarization.

### Scanning electron microscopy (SEM)

MOLT4 cells transfected for 72 h with Akt siRNA, RhoA siRNA, or scramble siRNA, or pretreated with 20 μM LY294002 for 2 h, were plated on poly-L-lysine coated coverslips and treated with 500 ng/ml Wnt5a for 3 h. After washing with PBS, cells were fixed with 2.5% glutaraldehyde/0.1 M sodium cacodylate buffer (pH 7.3) in PBS overnight at 4°C, then washed again with PBS. Cells were then fixed in 1% osmium tetroxide at 4°C for 2 h, washed thoroughly with distilled water, dehydrated by graded ethanol, and freeze-dried [22]. Specimens were sputter coated with platinum and observed with a scanning electron microscope (S-750; Hitachi, Tokyo, Japan) operating at 20 kV.

### Xenograft experiments

The animal care protocol was approved by the Medical Ethics Committee of Wuhan University School of Medicine (Permit Number: 14007). Animal studies were conducted in accordance with the regulations of the China Food and Drug Administration (CFDA) on Animal Care. Female SCID immunodeficient mice (aged 4 weeks) were purchased from Beijing HFK Bioscience Company, Ltd. Mice were maintained in an air-conditioned pathogen-free room under conditions of controlled lighting (12 h light/d) and fed a standard diet of laboratory food and water. 3×10^6^ MOLT4 cells were injected into SCID mice via the tail vein (n=3). The experimental groups received biweekly rWnt5A (75 ng/mouse) and/or CCL25 (30 ng/mouse) injections starting the third day after MOLT4 cell inoculation [14]. Mice were weighed every four days. Mice were anaesthetized 72 d post-inoculation, and MOLT4 cells in bone marrow and peripheral blood mononuclear cells (PBMCs) were detected by flow cytometry. Livers, lungs and spleens were removed and photographed.

### Immunohistochemistry

Mouse tissues were fixed in paraformaldehyde, embedded in paraffin, and sectioned (thickness = 5 μm). Following antigen retrieval, sections were incubated sequentially with 3% H_2_O_2_, goat serum, and anti-CD7 primary antibody (ABclonal, USA) overnight at 4°C. Thereafter, sections were incubated with HRP-conjugated secondary antibody for 30 min at 37°C. Diaminobenzidine (Sigma-Aldrich) was added for the chromogenic reaction, and cell nuclei were stained with hematoxylin. Sections were mounted and observed under an Olympus DP73 microscope at 400× magnification.

### Statistical analysis

Data were analyzed using Student's t test in GraphPad Prism 5 software. All data were expressed as means ± standard deviation (SD). For all analyses, a two-sided p-value <0.05 was deemed statistically significant.

## SUPPLEMENTARY MATERIALS FIGURES AND TABLES


