# Supplementary material for: Wnt5a and CCL25 promote adult T-cell acute lymphoblastic leukemia cell migration, invasion and metastasis
Source: Oncotarget. 2017 Mar 25;8(24):39033–47. doi: 10.18632/oncotarget.16559 (PMC5503593; doi:10.18632/oncotarget.16559)
Supplement: Supplementary file 1 [file oncotarget-08-39033-s001.pdf]

Wnt5a and CCL25 promote adult T-cell acute lymphoblastic leukemia cell migration, invasion and metastasis

SUPPLEMENTARY MATERIALS

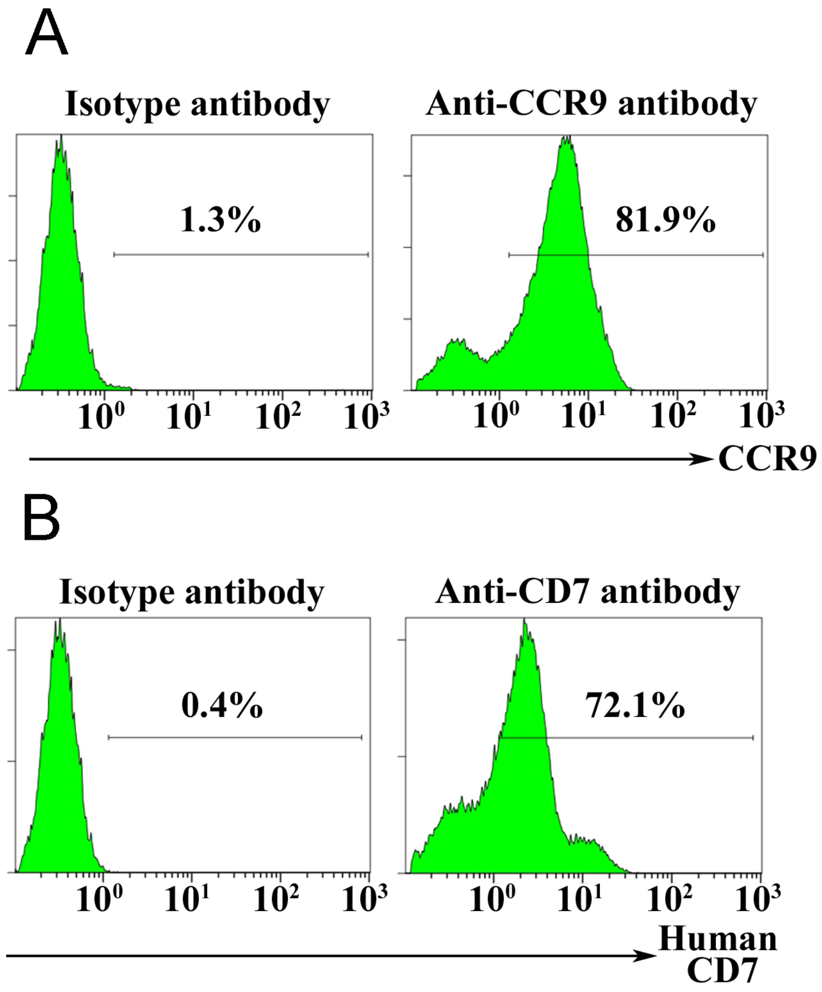

Supplementary Figure 1: Flow cytometry analyze the expression of CCR9 and CD7 in MOLT4 cells. MOLT4 cells were incubated with anti-human CD7 (Abclonal, USA) or FITC-labeled anti human CCR9 antibody (R&D Systems, Abingdon, United Kingdom) or matched isotype antibody (DAKO) in PBS containing 3% BSA and 0.1% sodium azide for 30 minutes, followed by washing three times with PBS, Dylight488-conjugated anti rabbit lgG antibody was followed for detecting CD7, after washing three times with PBS, cells finally resuspended in 300 µl PBS for flow cytometric analysis (Beckman, USA).

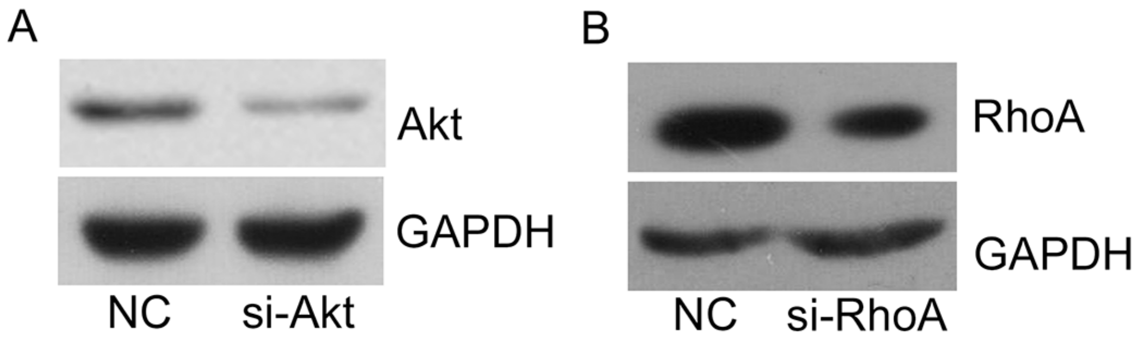

Supplementary Figure 2: Western blotting confirmed Akt and RhoA knockdown via siRNA in MOLT4 cells. MOLT4 cells transfected with Akt siRNA, RhoA siRNA, or scramble (NC) siRNA for 72 h, and Western blotting was used to confirm Akt **(A)** and RhoA **(B)** interference rate.

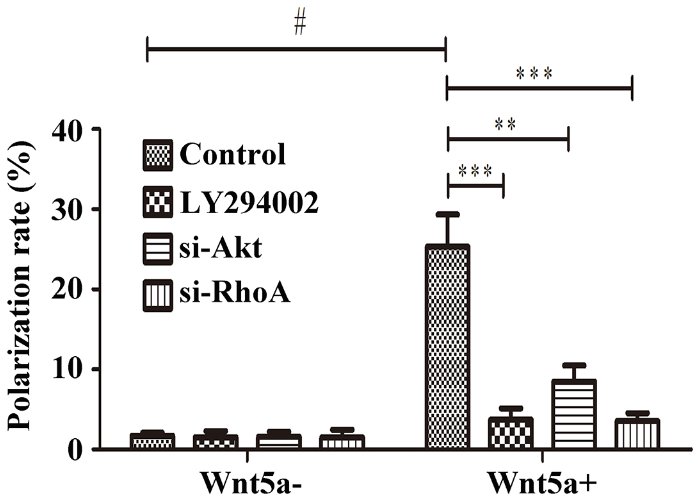

Supplementary Figure 3: The statistical figure of polarization ratio in MOLT4 cells. MOLT4 cells transfected with Akt siRNA, RhoA siRNA, or scramble (NC) siRNA for 72 h, or pretreated with 20 μM LY294002 for 2 h, were treated with 500 ng/ml Wnt5a for 3 h, and cell polarization was analyzed by LSCM.

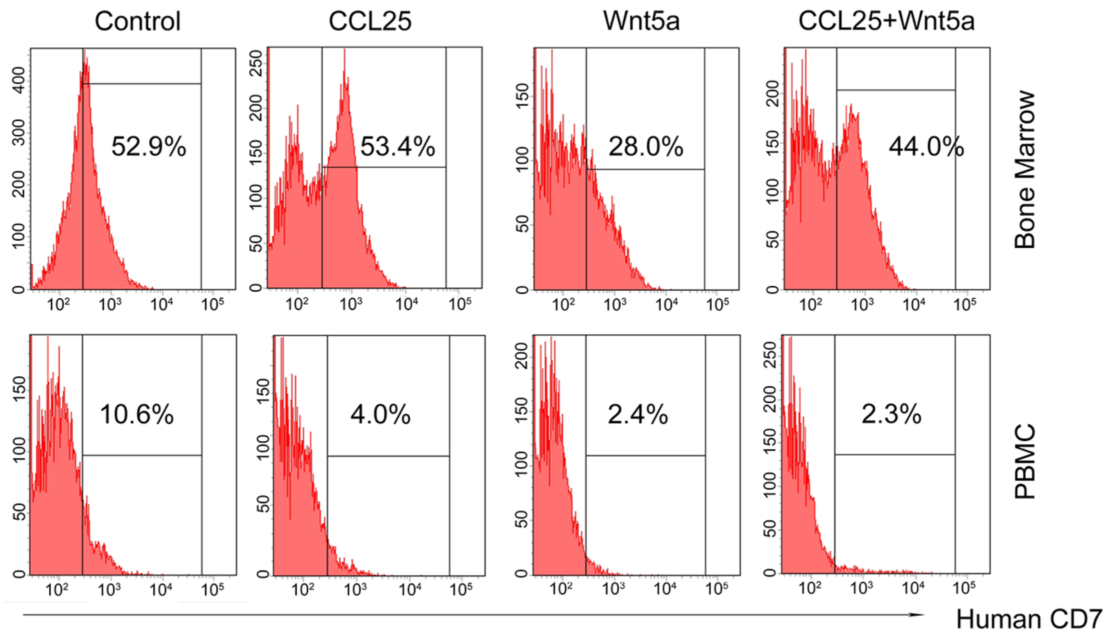

Supplementary Figure 4: MOLT4 cell ratio in bone marrow and PBMC. Leukocytes from the peripheral blood and bone marrow of different groups were separated by density gradient centrifugation using Ficoll-Paque Plus (GE Healthcare, Uppsala, Sweden) according to the manufacturer's instructions. Then, we detected the cell ratio of CD7 (the molecule marker of MOLT4 cells) by flow cytometry, just as Supplementary Figure 1.

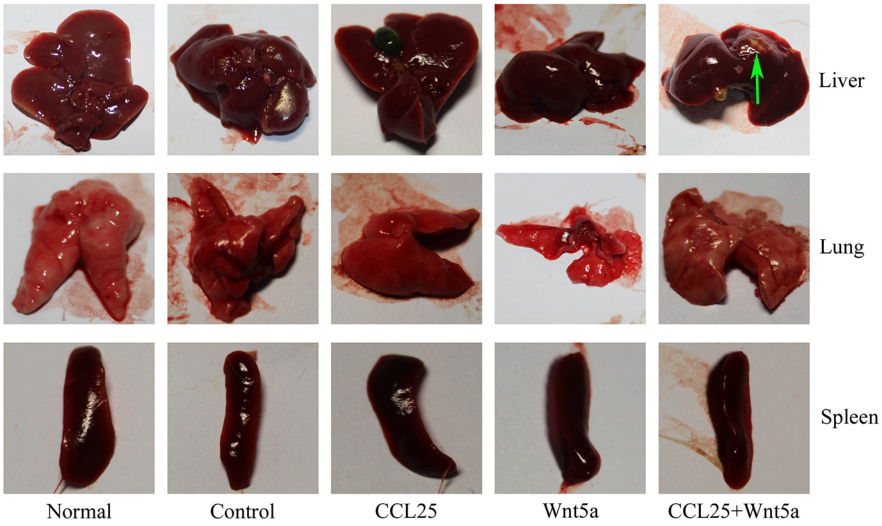

Supplementary Figure 5: The appearance of liver, lung and spleen of mice. Morphological observation of mice's liver, lung and spleen, arrow indicates tumor nodules.

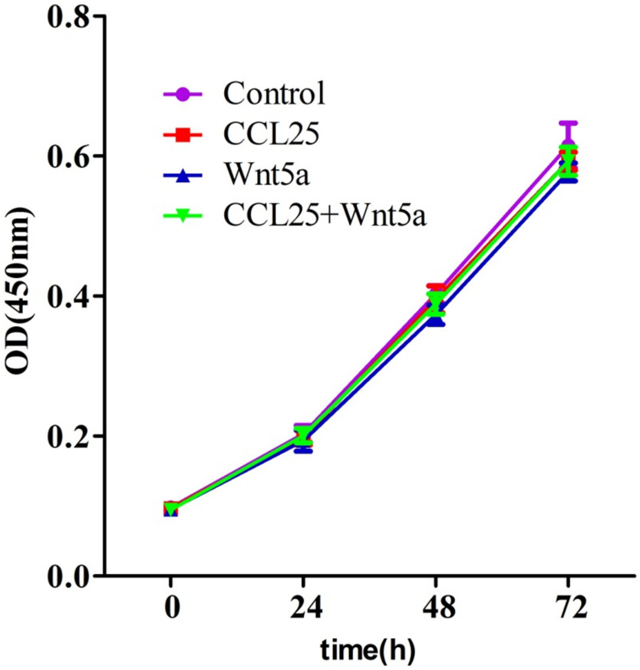

Supplementary Figure 6: The effects of Wnt5a and CCL25 on MOLT4 cell proliferation *in vitro*. MOLT4 cells treated with 100ng/ml CCL25 and/or 500ng/ml Wnt5a for indicated times, CCK8 was used to analyze the relative cell numbers at the end of experiment.

Supplementary Table 1: GSEA Analysis results (20 significant enrichment plots) using Wnt5a high (N=13) vs low (N=40) gene signatures obtained from adult T cell acute lymphocytic leukemia patient data (GSE42328)

GO TERM Biological Pathways	SIZE	ES	NES	NOM p-val	FDR q-val	FWER p-val
MONOOXYGENASE_ACTIVITY	29	0.53	1.82	0.00	0.14	0.10
REGULATION_OF_RAS_PROTEIN_ SIGNAL_TRANSDUCTION	19	0.72	1.76	0.00	0.22	0.26
GUANYL_NUCLEOTIDE_EXCHANGE_ FACTOR_ACTIVITY	46	0.68	1.70	0.00	0.32	0.41
RESPONSE_TO_ORGANIC_SUBSTANCE	30	0.61	1.67	0.00	0.36	0.53
REGULATION_OF_SMALL_GTPASE_ MEDIATED_SIGNAL_TRANSDUCTION	23	0.64	1.65	0.01	0.39	0.63
AUXILIARY_TRANSPORT_PROTEIN_ ACTIVITY	26	0.58	1.65	0.01	0.34	0.65
LAMELLIPODIUM	25	0.71	1.64	0.00	0.32	0.67
REGIONALIZATION	15	0.66	1.64	0.01	0.29	0.67
ACTIVATION_OF_NF_KAPPAB_ TRANSCRIPTION_FACTOR	18	0.68	1.62	0.01	0.34	0.74
PATTERN_SPECIFICATION_PROCESS	31	0.54	1.60	0.01	0.40	0.81
ACTIN_CYTOSKELETON_ ORGANIZATION_AND_BIOGENESIS	105	0.51	1.59	0.00	0.37	0.81
OXIDOREDUCTASE_ACTIVITY_ GO_0016705	35	0.55	1.59	0.02	0.35	0.81
LIPID_TRANSPORTER_ACTIVITY	28	0.64	1.57	0.01	0.43	0.87
CHANNEL_REGULATOR_ACTIVITY	24	0.53	1.56	0.02	0.46	0.89
ACTIN_FILAMENT_ORGANIZATION	24	0.69	1.55	0.01	0.46	0.91
REGULATION_OF_GTPASE_ACTIVITY	15	0.69	1.52	0.03	0.61	0.96
GOLGI_ASSOCIATED_VESICLE	28	0.61	1.52	0.02	0.60	0.96
PHOSPHOINOSITIDE_BINDING	20	0.69	1.50	0.02	0.64	0.97
CARBOHYDRATE_BIOSYNTHETIC_ PROCESS	49	0.54	1.50	0.02	0.62	0.97
ELECTRON_TRANSPORT_GO_0006118	51	0.53	1.50	0.02	0.59	0.97

Supplementary Table 2: PCR primers

Gene	Forward primer(5’-3’)	Reverse primer(5’-3’)
GAPDH	CTGGG CTACACTGAGCACC	AAGTGGTCGTTGAGGGCAATG
Wnt5a	ATTCTTGGTGGTCGCTAGGTA	CGCCTTC TCCGATGTACTGC
Wnt1	AGGTTCCATCGAATCCTGCAC	CATCTCGGAGAATACGGTCGT
Wnt2	CCTGATGAATCTTCACAACAACAGA	TCCCACAGCACATGACTTCACAG
Wnt2b	TGCTGTACGGTGCAAGGAAT	AATAGAAGCGGTAGCAGCCC
Wnt3	CTCGCTGGCTACCCAATTTG	AGGCTGTCATCTATGGTGGTG
Wnt4	CTCGTGCCTGCGTTCG	CCTTGGACGTCTTGTTGCAT
Wnt5a	CTTCGCCCAGGTTGTAATTGAAGC	CTGCCAAAAACAGAGGTGTTATCC
Wnt5b	GAAGCTGTGCCAATTGTACCAG	GCTGCCTATCTGCATGACTCTCC
Wnt6	ATGCTGCCGCCCTTACCC	CACAGGCAGAGGCTGAGCT
Wnt7a	TGGGCGCAAGCATCATCTGTAAC	CATCCACAAAGACCTTGGCGAA
Wnt7b	TCCTGTACGTGAAGCTCGGA	TCGGCTTGGTTGTAGTAGCC
Wnt8a	GCTTGGGAACGCTGGAACTGC	CATTGTTTGACCCATCACAGCCAC
Wnt8b	CAGTGCCAATCGGGAGACAGC	GACAAACTGCTTGGAAATCGCC
Wnt9a	AAGCGAGGCTTCAAGGAGAC	CCTTGACGAACTTGCTGCTG
Wnt9b	GTTTCAGTTCCGGCATGAGC	CCTGAGGCCACTCTTCACAG
Wnt10a	CACAGTGTGCCTAACATTGCCAG	CTGCTGAAGATGGGACTCTCATAG
Wnt10b	CATCCAGGCACGAATGCGA	CGGTTGTGGGTATCAATGAAGA
Wnt11	CCATGGAGCTCTGCTTGTGA	CTCCTTACACCAGCCTGTGG
Wnt16	TTCAGACACGAGAGATGGAACT	CCAGCCTTCACTTGCTGAG
